# Glucose-Uptake Activity and Cytotoxicity of Diterpenes and Triterpenes Isolated from Lamiaceae Plant Species

**DOI:** 10.3390/molecules25184129

**Published:** 2020-09-10

**Authors:** Ninon G. E. R. Etsassala, Kadidiatou O. Ndjoubi, Thilly J. Mbira, Brendon Pearce, Keenau Pearce, Emmanuel I. Iwuoha, Ahmed A. Hussein, Mongi Benjeddou

**Affiliations:** 1 Department of Horticultural Sciences, Cape Peninsula University of Technology, Symphony Rd., Bellville 7535, South Africa; ninonetsassala@yahoo.fr; 2 Chemistry Department, Cape Peninsula University of Technology, Symphony Rd., Bellville 7535, South Africa; dickakadi@yahoo.fr (K.O.N.); tilly.mbira@gmail.com (T.J.M.); mohammedam@cput.ac.za (A.A.H.); 3 Precision Medicine Laboratory, Department of Biotechnology, 2nd Floor, Life Science Building, University of the Western Cape, Cape Town 7530, South Africa; brendon.biff@gmail.com (B.P.); keenau.pearce@gmail.com (K.P.); 4 Chemistry Department, University of the Western Cape, Private Bag X17, Bellville 7535, South Africa; eiwuoha@uwc.ac.za

**Keywords:** diabetes mellitus, *Lamiaceae*, terpenoids, abietane diterpenes, ursolic acid, glucose uptake, toxicity, insulin resistance

## Abstract

The prevalence of diabetes mellitus (DM), considered one of the most common metabolic disorders, has dramatically increased and resulted in higher rates of morbidity and mortality around the world in the past decade. It is well known that insulin resistance in target tissues and a deficiency in insulin secretion from pancreatic β-cells are the main characteristics of type 2 diabetes. The aim of this study was the bio-evaluation of compounds isolated from three selected plant species: namely, *Salvia africana-lutea*, *Leonotis ocymifolia*, and *Plectranthus madagascariensis*, for their glucose-uptake ability. Methanolic extracts were produced from the aerial parts of each plant. Compounds were identified using different spectroscopic techniques. The glucose-uptake ability of each compound was then evaluated in mammalian cells using 2-deoxyglucose-6-phosphate. The cytotoxicity of each compound was established via the MTT assay. Chromatographic purification of the three plant species yielded sixteen pure terpenoids. Compounds **1** (*p* = 0.0031), **8** (*p* = 0.0053), and **6** (*p* = 0.0086) showed a marked increase in glucose uptake, respectively. Additionally, **1**, **4**, and **6** exhibited cytotoxicity toward mammalian tissue with a decrease in cell viability of ~70%, ~68%, and ~67%, respectively. The results suggested that several compounds demonstrated a marked increase in glucose uptake, while two of the compounds exhibited signs of cytotoxicity. It may, therefore, be suggested that these compounds be considered as potential candidates for novel plant-derived alternative therapies in the treatment of type 2 diabetes.

## 1. Introduction

Diabetes mellitus (DM) is considered as one of the most common metabolic disorders with high rates of morbidity and mortality around the world [1]. It is well known that insulin is vital in glucose homeostasis, as it stimulates the transport of blood glucose within the skeletal muscle [2]. However, insulin resistance in target tissues and deficiency of insulin secretion from pancreatic β-cells are the main characteristics of type 2 diabetes. In addition, characterization of DM also includes a decrease in peripheral glucose uptake into muscle, adipose, or liver cells as well as an increase in endogenous glucose production, causing the increase in blood glucose concentration [3,4,5]. Therefore, agents with a capacity to stimulate glucose uptake in these tissues can be used to improve insulin resistance and consequently treat diabetes [6]. A huge number of synthetic antidiabetic agents, such as acarbose, miglitol, sulfonylurea, metformin, and thiozolidinedione, are readily available in the market. However, their effectiveness is limited due to high cost and adverse side effects [7,8], with the exception of the widespread use of metformin due to its relatively low cost. Therefore, there is a great need to develope potent natural antidiabetic products of high-safety margin. Furthermore, selected species from the Lamiaceae family have long been used to treat a plethora of ailments. These include *Leonotis ocymifolia,* traditionally used in Ethiopia for the treatment of diabetes [9]; *Plectranthus madagascariensis*, used for scabies and small wounds as well as in the treatment of colds, asthma, cough, and chest complaints [10], also reported to be an effective inhibitor of alpha-glucosidase [11], and *Salvia africana-lutea*, which is traditionally used for the treatment of different kinds of ailments and/or diseases, such as coughs, sexual debility, mental, and nervous conditions, throat inflammation, chronic bronchitis, tuberculosis, influenza, stomach-ache, diarrhea, and urticaria [12]. It has been reported to be a rich source of terpenoids with potent alpha-glucosidase and alpha-amylase inhibitory activities [8]. Numerous terpenoids isolated from Lamiaceae, such as ursolic acid, (**8**) have been reported to stimulate glucose uptake in adipocytes through the phosphatidylinositol (PI) 3-kinase (PI3K) pathway and enhance glucose transporter 4 (GLUT4) translocation and expression [13]. In addition, Oleanolic acid (**7**) and its derivatives have also been reported to up-regulate the expression of GLUT4, which increases the glucose-uptake activity in adipose and muscle cell lines [14,15]. Carnosic acid and carnosol also activate Akt and AMPKα signaling and then enhance glucose uptake in L6 myotubes as well as stimulate glucose uptake in L6 myotubes [16,17,18].

The present study primarily examines the glucose-uptake activity as well as the cytotoxicity of different phytochemical constituents isolated from three different plant species of Lamiaceae family: *S. africana-lutea, P. madagascariensis*, and *L. ocymifolia.*

## 2. Results

**Figure 1:**
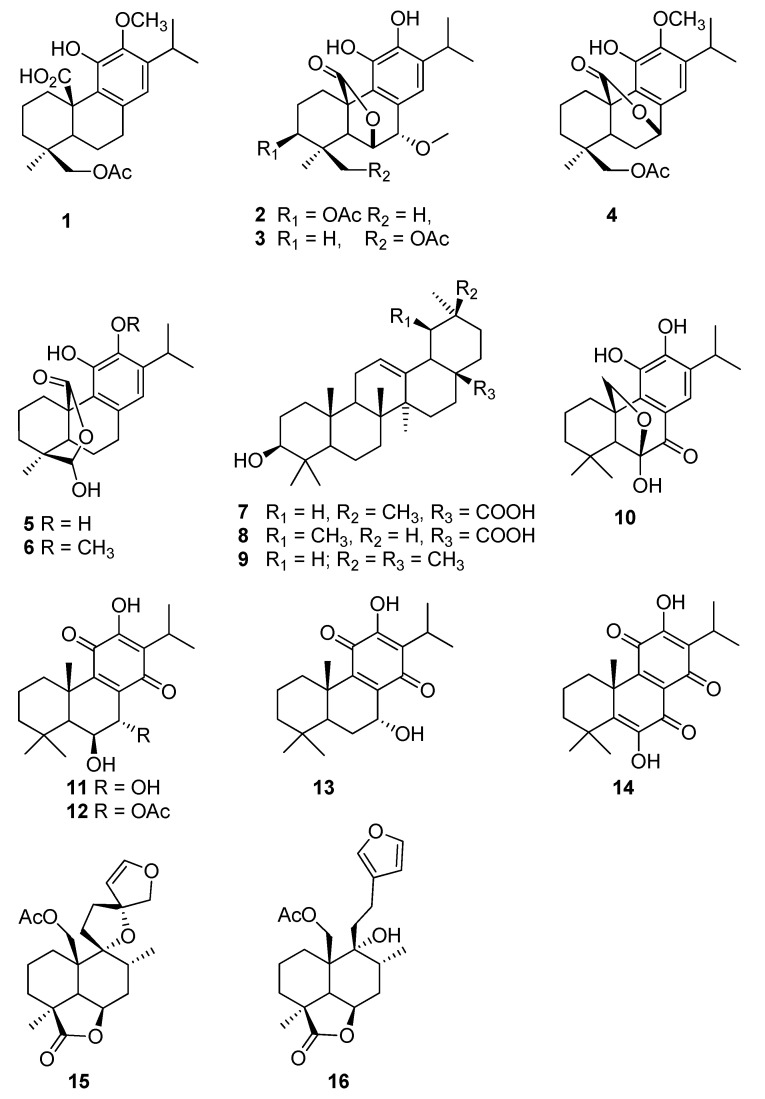
Chemical structures of the isolated compounds.

Sixteen terpenoids (Figure 1), including thirteen diterpenes and three triterpenes, were purified from three Lamiaceae species, *S. africana-lutea*, *P. madagascariensis*, and *L. ocymifolia* and tested for their ability to regulate the glucose intake in HEK293 kidney cells line.

From *S. africana lutea*, six abietane diterpenes were isolated and identified as 19-acetoxy-12-methoxycarnosic acid (**1**), 3β-acetoxy-7α-methoxyrosmanol (**2**), 19-acetoxy-7α-methoxyrosmanol (**3**), 19-acetoxy-12-methoxy carnosol (**4**), clinopodiolides A (**5**), and B (**6**), in addition to three triterpenes, oleanolic and ursolic acids (**7**, **8**) and β-amyrin (**9**) [19,20,21,22,23,24].

The phytochemical analysis of *P. madagascariensis* total extract resulted in the isolation of five known compounds namely carnosolon (**10**), 6β,7α-dihydroxyroyleanone (**11**), 7α-acetoxy-6β-hydroxyroyleanone (**12**), horminone (**13**), and coleon U quinone (**14**). The NMR data of the isolated abietane diterpenoids were compared to that of previously isolated constituents from the plant and other species of the genus *Plectranthus* [25,26,27,28].

From *L. ocymofolia*, two labdane diterpenes were isolated and identified as Leonurun (**15**) and 20-acetoxy-marrubiin (**16**) [29,30].

**Figure 2:**
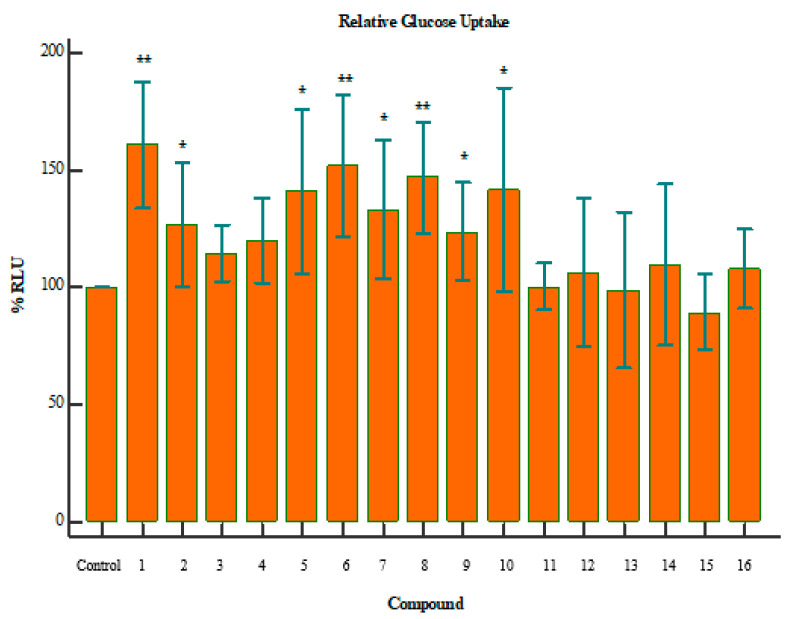
Relative glucose uptake of compounds **1**–**16**; where the *p*-value is indicative of the statistical significance versus the control, using an independent two-tailed T-test. * *p* < 0.05, ** *p* < 0.01. Compounds 1 (*p* = 0.0031), 8 (*p* = 0.0053), and 6 (*p* = 0.0086) showed a marked increase in glucose uptake.

The results demonstrated that when cells are exposed to 2-deoxyglucose (2DG), there are transported across the membrane and rapidly phosphorylated in the same manner as glucose. However, enzymes that further modify glucose-6-phosphate (G6P) cannot modify 2DG6P, and thus, a membrane-impermeable analyte accumulates in the cell. After a brief period of incubation, the acidic Stop Buffer is added to lyse cells, terminates uptake and destroys any NADPH. A high-pH buffer solution (neutralization buffer) is then added to neutralize the acid. A detection reagent is added to the sample wells. Glucose-6-phosphate dehydrogenase oxidizes the deoxyglucose to 6-phosphodeoxygluconate and simultaneously reduces NADP+ to NADPH. The reductase uses NADPH to convert the proluciferin to luciferin, which is then used by Ultra-Glo™ recombinant luciferase to produce a luminescent signal that is proportional to the concentration of 2DG6P. Figure 2 indicates the relative glucose uptake for a given compound, compared to the control. The *p*-values in these graphs were calculated using independent two-tailed T-test, where 0.05 is the threshold for significance.

## 3. Discussion

With the global rise in the cost of medicines, many are turning to alternative forms of treatment. Herbal medicine is traditionally used in many cultures globally as a more cost-effective method of treatment. The data presented herein aid in confirming the usefulness of specific plant-derived compounds (phytochemicals) in the treatment of diabetes mellitus [31,32].

*Plectranthus madagascariensis* has been reported to be an effective inhibitor of alpha-glucosidase and a promising source of secondary metabolites with significant alpha-glucosidase inhibitory activity. Three abietane diterpenoids, such as 6β,7α-dihydroxyroyleanone (**11**), 7α-acetoxy-6β-hydroxyroyleanone (**12**), coleon U quinone (**14**), in addition to rosmarinic acid, were isolated from methanol extract of *P. madagascariensis* and exhibited alpha-glucosidase inhibitory activity with IC_50_ values of 274.9 ± 12.3, 108.2 ± 1.3, 142.7 ± 1.4 μmol/L, and 33.0 ± 4.6 μmol/L, respectively [10].

Etsassala et al. [8] have reported on the in vitro bio-evaluation of terpenes isolated from *Salvia africana lutea* against alpha-glucosidase and alpha-amylase. The results showed strong inhibitory activities for **8**, **10**, and **7** with IC_50_ values of 11.3 ± 1.0, 17.1 ± 1.0, and 22.9 ± 2.0 µg/mL, respectively. Compound **7** demonstrated the strongest in vitro alpha-amylase inhibitory activity among the tested compounds with IC_50_ of 12.5 ± 0.7 µg/mL, followed by compounds 8 and 10 with IC_50_ values of 66.1 ± 2.0 µg/mL and 76.6 ± 2.1 µg/mL, respectively. [8]. Other studies also confirmed the bioactivity demonstrated by **7** and **8 [33]**. Leonurun (**15**) and 20-acetoxy-9α,l3-dihydroxy-15(16)-epoxylabd-14-en-6β(19)-lactone (**16**) were not active against alpha-glucosidase and amylase comparing with other compounds when tested up to 50 µg/mL.

Compounds **1**, **8**, and **6** showed a marked increase in glucose uptake (Figure 2). The exact mechanism of this action is yet to be derived, but it is suspected that these compounds increase glucose sensitivity through stimulation of glucose metabolism. In addition, it may be concluded that these compounds could potentially aid in the treatment of diabetes mellitus. Compound **8** has been reported to stimulate glucose uptake in 3T3-L1 adipocytes through the PI3K pathway. Additionally, **8** has also been reported to lower blood glucose and improve insulin resistance and diabetes which corroborate with our findings [13,33,34]. **7** has been reported to up-regulate the expression of GLUT4, which increases the glucose-uptake activity in adipose and muscle cell lines [14,35].

However, it is important to note that the raw compound may not be effectively taken up by the cells [36]. This could be influenced by a number of factors including the number of surviving cells after treatment, the solubility of the compound, binding affinity between the compound and the cell membrane, and the length of treatment incubation [36].

Furthermore, although a marginal increase in glucose uptake was seen in the remaining compounds, the statistical significance of those findings is lacking. It may be suggested that they are more effective in combination with other compounds, or that they simply have an even weaker cellular uptake efficiency. Nonetheless, further investigation will be required to define the capabilities of these compounds.

**Figure 3:**
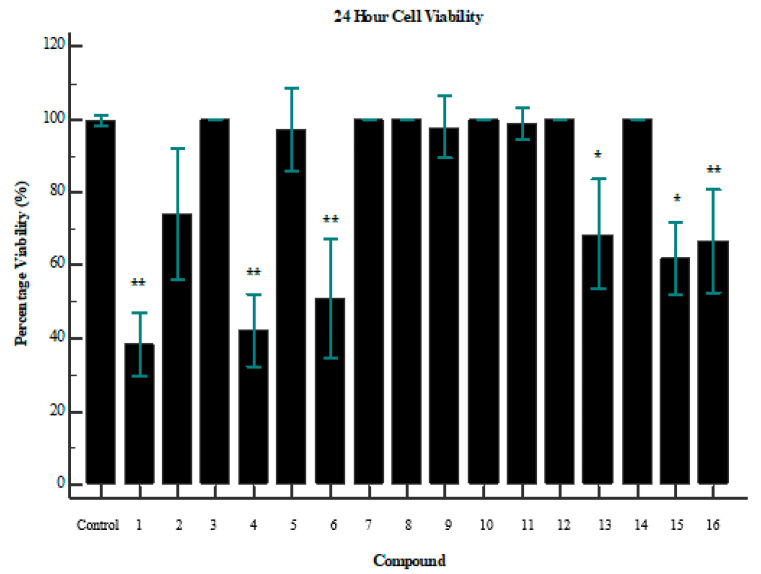
Cell viability of tested compounds after 24 h; where the *p*-value is indicative of the statistical significance versus the control, using an independent two-tailed T-test (*n* = 3). * *p* < 0.05, ** *p* < 0.01. Compounds 1 (*p* = 0.0056), 4 (*p* = 0.0017), and 6 (*p* = 0.0060) showed the greatest impact on cell viability.

Interestingly, an overlap between glucose uptake and cytotoxicity exists for some of the compounds. Compounds **1** and **6** both improved glucose uptake and showed signs of cytotoxicity (Figure 3). Compound **1** reduced cell viability by ~70% (*p* = 0.0056), while **6** reduced cell viability by ~52% (*p* = 0.0060). However, it is important to note the difference in dosage between the assays. Cell-viability assays were performed with a concentration of 250 µg/mL, while glucose-uptake assays were performed with a maximum dose of 100 µg/mL. It is, therefore, confirmed that beyond a dosage of 100 µg/mL the compound becomes toxic to the cells. This is a crucial consideration when evaluating compounds as potential drug targets. Further investigation of the minimum usable dosage for improving glucose uptake is required, as well as the minimum dosage that causes toxicity in a larger collection of cell types.

## 4. Materials and Methods

### 4.1. Chemicals and Reagents

Organic solvents, such as methanol (HPLC grade), ethanol, ethyl acetate, and hexane, were supplied by Merck (Cape Town, South Africa). Thin layer chromatography (TLC) was performed on normal-phase (Merck) Silica gel 60 PF_254_ pre-coated aluminum plates. Column chromatography was conducted on silica gel 60 H (0.040–0.063 mm particle size, Merck, Cape Town, South Africa) and Sephadex LH-20 (Sigma-Aldrich, Cape Town, South Africa).

NMR spectra were recorded on an Avance 400 MHz NMR spectrometer (Bruker, Rheinstetten, Germany)/Varian 200 MHz Mercury, in deuterated chloroform and acetone, using the solvent signals as the internal reference. HRMS analysis was conducted on an Ultimate 3000 LC (Dionex, Sunnyvale, CA, USA) coupled to a Bruker QTOF with an electrospray ionization (ESI) interface working in the positive ion mode. Preparative HPLC was used for further isolation of pure compounds using HPLC methanol and distilled water.

Renocytes (HEK293 kidney cells) were obtained from the American Type Culture Collection (Manassas, VA, USA) and cultured in DMEM containing essential amino acids, sodium pyruvate, and l-glutamine. Cell seeding was done on 24-well plates (50,000 cells/well) for the 2-deoxy-[^3^H]-D-glucose assay.

### 4.2. Plant Material

*Salvia africana-lutea* and *Leonotis ocymifolia* aerial parts were collected in May, 2018, from Cape Flats Nature Reserve, University of the Western Cape, and *Plectranthus madagascariensis* was collected in February 2019 from Cape Peninsula University of Technology, Bellville campus. The identification of the plants was carried out by Prof. Christopher Cupido (Department of Botany, Fort Hare University, South Africa), with herbarium numbers NBG1465544-0, NBG1465551-0, and NBG1465552-0 respectively.

### 4.3. Extraction and Purification of Chemical Constituents

Compounds **1**–**9** were available in the lab from previous study and isolated from *S. africana lutea* [8]. Compounds **10**–**14** were isolated from *P. madagascariensis* as follows: The aerial parts of *P. madagascariensis* were extracted with DCM-MeOH (3:1) and the total extract (13.0 g) was subjected to silica gel column chromatography using Hex/EtOAc gradient of increasing polarity to yield 16 main fractions. The main fraction III (90.8 mg) was subjected to isocratic column chromatography using Hex/EtOAc gradient (90:10) to yield **13** (13.5 mg). Main fraction VI (200 mg) was subjected to sephadex LH-20 using 95% methanol (MeOH) and 5% deionized water (DW), then isocratic silica gel column chromatography using Hex/EtOAc gradient (98:2) to yield **12**. (61.9 mg). The main fraction VIII (97 mg) was applied to a sephadex LH-20 (5% aqueous MeOH) to produce **11** (25.7 mg). Main fraction XI (62.21 mg) was subjected to sequential Sephadex LH-20 (5% aqueous MeOH), then HPLC using gradient of acetonitrile/DW (60% to 80% in 30 min, then 100% acetonitrile for 15 min) to produce **14** (R_t_ 39.5 min, 6 mg). Main fraction XIII and XIV (400 mg) were combined and chromatographed to Sephadex LH-20 using MeOH/DW (95:5) to produce **10** (26.7 mg).

Compounds **15** and **16** were isolated from *Leonotis ocymifolia* var. *raineriana* as follows: The fresh plant materials of (1.0 kg) were blended and extracted with methanol (4.5 L), and after filtration and solvent evaporation, the extract (42.0 g) was loaded on silica gel column and eluted using gradient of Hex/EtOAc in order of increasing polarity. Fraction 12 yielded crystals, which was identified as compound **15**. Fraction 20 after fractionation using silica gel column Hex: EtOAc (80: 20 to 70:30) gave compound **16**.

### 4.4. Spectroscopic Data of Compounds **10**–**16**

**Compound 10.**^1^H-NMR (400 MHz, CDCl_3_), δ_H_ 7.65 (s, H-14), 4.29 (d, *J* = 7.5 Hz, H_β_-20), 3.37 (d, *J* = 7.5 Hz, H_α_-20), 3.02, (sept, *J* = 7.1 Hz, H-15), 1.31 (s, Me-19), 1.17 (d, *J* = 7.1 Hz, H-16), 1.16 (d, *J* = 7.1 Hz, H-17), 1.04 (s, Me-18). ^13^C-NMR (100 MHz, CDCl_3_), δ_C_ 192.8 (C-7), 148.3 (C-12), 140.5 (C-11), 137.7 (C-9), 133.3 (C-13), 120.1 (C-14), 121.4 (C-8), 105.2 (C-6), 72.0 (C-20), 58.2 (C-5), 51.47 (C-10), 41.33 (C-3), 33.7 (C-18), 32.4 (C-4), 29.6 (C-1), 27.1 (C-15), 22.5 (C-17), 22.4 (C-16), 22.2 (C-19), 18.5 (C-2).

**Compound 11.**^1^H-NMR (400 MHz, CDCl_3_), δ_H_ 4.53 (d, *J* = 1.5 Hz, H-7), 4.46 (br s, H-6), 3.18 (sept, *J* = 7.1 Hz, H-15), 1.62 (s, Me-20), 1.27 (s, Me-19), 1.23 (d, *J* = 7.1 Hz, H-16), 1.23 (d, *J* = 7.1 Hz, H-17), 1.06 (s, Me-18). ^13^C-NMR (100 MHz, CDCl_3_), δ_C_ 189.5 (C-14), 183.1 (C-11), 151.2 (C-12), 147.6 (C-9), 140.9 (C-8), 124.3 (C-13), 69.3 (C-6), 69.1 (C-7), 49.5 (C-5), 42.3 (C-3), 38.6 (C-10), 38.4 (C-1), 33.7 (C-4), 33.5 (C-18), 24.3 (C-19), 24.0 (C-15), 21.6 (C-20), 19.9 (C-17), 19.8 (C-16), 19.0 (C-2).

**Compound 12.**^1^H-NMR (400 MHz, CDCl_3_), δ_H_ 5.60 (d, *J* = 1.8 Hz, H-7), 4.24 (s, H-6), 3.09 (sept, *J* = 7.1 Hz, H-15), 1.98 (s, CH_3_CO), 1.55 (s, Me-20), 1.16 (s, Me-19), 1.13 (d, *J* = 7.1 Hz, H-16), 1.11 (d, *J* = 7.1 Hz, H-17), 0.92 (s, Me-18). ^13^C-NMR, δ_C_ (100 MHz, CDCl_3_) 186.0 (C-14), 183.1 (C-11), 151.2 (C-12), 150.1 (C-9), 137.0 (C-8), 124.3 (C-13), 69.0 (C-7), 66.4 (C-6), 49.7 (C-5), 42.33 (C-3) 38.6 (C-4) 38.3 (C-1), 33.6 (C-10), 33.5 (C-18), 18.9 (C-2), 24.1 (C-15), 23.6 (C-19), 21.3 (C-20), 19.8 (C-17), 19.6 (C-16), 21.0/170.1 (CH_3_CO).

**Compound 13.**^1^H-NMR (400 MHz, CDCl_3_), δ_H_ 4.73 (d, *J* = 1.5 Hz, H-7), 3.16 (sept, *J* = 7.1 Hz, H-15), 1.22 (s, Me-20), 1.21 (d, *J* = 7.1Hz, H-16), 1.20 (d, *J* = 7.1 Hz, H-17), 0.98 (s, Me-18), 0.90 (s, Me-19). ^13^C-NMR (100 MHz, CDCl_3_) δ_C_ 189.2 (C-14), 183.9 (C-11), 151.1 (C-12), 147.8 (C-9), 143.3 (C-8), 124.2 (C-13), 63.2 (C-7), 45.8 (C-5), 41.13 (C-3), 39.8 (C-10), 35.8 (C-1), 33.2 (C-4), 33.1 (C-18), 25.8 (C-6), 24.0 (C-15), 21.7 (C-19), 19.9 (C-16), 19.8 (C-17), 19.0 (C-2), 18.4 (C-20).

**Compound 14.**^1^H-NMR (400 MHz, CDCl_3_), δ_H_ 3.22 (sept, *J* = 7.0 Hz, H-15), 1.64 (s, Me-20), 1.43 (s, Me-18), 1.42 (s, Me-19), 1.25 (d, *J* = 7.1 Hz, H-16), 1.24 (d, *J* = 7.0 Hz, H-17). ^13^C-NMR (100 MHz, CDCl_3_), δ_C_ 184.3 (C-14), 183.6 (C-11), 177.5 (C-7), 155.1 (C-9), 150.7 (C-12), 146.8 (C-6), 143.3 (C-5), 126.8 (C-8), 126.0 (C-13), 41.4 (C-10), 36.4 (C-4), 36.3 (C-3), 30.8 (C-1), 29.1 (C-19), 27.5 (C-20), 27.2 (C-18), 24.4 (C-15), 19.8 (C-16), 19.8 (C-17), 17.7 (C-2).

**Compound 15.**^1^H-NMR (200 MHz, CDCl_3_), δ_H_ 6.34 (br d, *J*= 2.6 Hz, H-15), 5.04 (br d, *J*= 2.6 Hz, H-14), 4.59 (br t, *J*= 4.0 Hz, H-6), 4.26 and 4.17 (d each, *J*= 12.5 Hz, CH_2_-20), 4.28 and 3.95 (*d*, each, *J* = 10.6 Hz, H-16a, b), 1.12 (s, Me-18), 0.73 (d, *J* = 6.4 Hz, Me-17). ^13^C-NMR (50 MHz CDCl_3_) δ_C_ 182.3 (C-19), 147.9 (C-15), 107.0 (C-14), 91.3 (C-13), 88.7 (C-9), 79.7 (C-16), 75.5 (C-6), 65.6 (C-20), 46.4 (C-5), 43.2 (C-4), 42.0 (C-10), 37.0 (C-12), 32.4 (C-8), 31.5 (C-7), 31.4 (C-11), 28.0 (C-3), 23.4 (C-18), 22.2 (C-1), 17.6 (C-2), 17.4 (C-17), 20.7/169.8 (COCH_3_).

**Compound 16.**^1^H-NMR (200 MHz, CDCl_3_) δ_H_ 7.34 (br s, H15), 7.21 (br s, H-16), 6.24 (br s, H-14), 5.13 (br s, H-6), 4.27/4.62 (br d each *J*= 12.3 Hz, CH_2_-20), 1.01 (s, Me-18), 0.96 (d, *J* = 6.3 Hz, Me-17). ^13^C-NMR (50 MHz, CDCl_3_) δ_C_ 175.9 (C-19), 143.4 (C-15), 138.4 (C-16), 124.2 (C-13), 110.3 (C-14), 75.8 (C-20), 74.7 (C-9), 69.3 (C-6), 46.5 (C-5), 43.9 (C-11), 41.0 (C-10), 40.8 (C-4), 39.6 (C-3), 33.3 (C-8), 30.1 (C-1), 32.8 (C-7), 22.3 (C-18), 22.4 (C-2), 20.3 (C-12), 15.6 (C-17), 20.9/170.2 (COCH_3_).

### 4.5. Glucose-Uptake Assay

The method for measuring glucose uptake in mammalian cells was based on the detection of 2-deoxyglucose-6-phosphate and was performed according to the manufacturer’s guidelines listed in the Table 1. When cells are exposed to 2-deoxyglucose (2DG), there are transported across the membrane and rapidly phosphorylated in the same manner as glucose. However, enzymes that further modify glucose-6-phosphate (G6P) cannot modify 2DG6P, and thus, a membrane-impermeable analyte accumulates in the cell. After a brief period of incubation, the acidic stop buffer is added to lyse cells, terminates uptake and destroys any NADPH. A high-pH buffer solution (neutralization buffer) is then added to neutralize the acid. A detection reagent is added to the sample wells. Glucose-6-phosphate dehydrogenase oxidizes the deoxyglucose to 6-phosphodeoxygluconate and simultaneously reduces NADP+ to NADPH. The reductase uses NADPH to convert the proluciferin to luciferin, which is then used by Ultra-Glo™ recombinant luciferase to produce a luminescent signal that is proportional to the concentration of 2DG6P.

The reaction mixtures were incubated at room temperature for 1 h. After 1 h, the cells were incubated with various concentrations of each compound, and the cells were washed with 100µl PBS. A volume of 50 µL 1 mM 2DG was added to each well and allowed to incubate for 10 min. A volume of 25 µL stop buffer was added to each well and shaken briefly. Thereafter, 25 µL of neutralization buffer was added to each well and shaken briefly. Finally, a volume of 100 µL of reductase substrate mix was added and the plate shaken briefly. The plate was then incubated at room temperature for 30 min and read on a plate reader at 15 min intervals for 2 h.

### 4.6. Cytotoxicity Assay (MTT)

The cytotoxic effect of each compound on human embryonic kidney (HEK293) cells was assessed following the well-established MTT protocol. Cells were cultured in DMEM containing essential amino acids, sodium pyruvate, and L-glutamine at 37 °C in 96-well microtiter plates (10,000 cells/well). The plates were exposed to a dose of 250 µg of each compound for 24 h. Untreated cells served as control. After treatment, the medium was separated, and cells were incubated with 200 µL of MTT in fresh medium at 37 °C for 4 h. The resultant formazan crystals from the mitochondrial reduction in MTT were solubilized in DMSO. The absorbance of each sample was determined using a microplate absorbance reader at 570 nm, and the percentage of cell viability was calculated using the following equation: cell viability (%) = (absorbance of test – absorbance of background/absorbance of control – absorbance of background) × 100, according to manufacturer instructions (Dojindo, Maryland, MD, USA).

## 5. Conclusions

This present work is the first scientific report on the investigation of the glucose-uptake activity and cytotoxicity of abietane and labdane diterpenes and triterpenes isolated from selected plants species from Lamiaceae family, and the results suggested that **1**, **8**, and **6** showed a marked increase in glucose uptake, while **1** and **6** exhibited signs of cytotoxicity. Oleanolic (**7**) and ursolic (**8**) acids have been reported to increase the glucose uptake using several bioassays [13,14,15]; however, in this report, ursolic acid (**8**) exhibited more significant activity over oleanolic acid (**7**). It may, therefore, be suggested that ursolic acid be considered as a potential candidate for novel plant-derived alternative therapies in the treatment of type 2 diabetes.

## Figures and Tables

**Table 1:** Constituents of glucose-uptake assay.

Reagent	1 Reaction (µL)	50 Reactions (µL)
Luciferase reagent	100	5000
NADP+	1	50
G6PDH	2.5	125
Reductase	0.5	25
Reductase substrate	0.0625	3
